# Gradual Loss of ACTH Due to a Novel Mutation in *LHX4*: Comprehensive Mutation Screening in Japanese Patients with Congenital Hypopituitarism

**DOI:** 10.1371/journal.pone.0046008

**Published:** 2012-09-24

**Authors:** Masaki Takagi, Tomohiro Ishii, Mikako Inokuchi, Naoko Amano, Satoshi Narumi, Yumi Asakura, Koji Muroya, Yukihiro Hasegawa, Masanori Adachi, Tomonobu Hasegawa

**Affiliations:** 1 Department of Pediatrics, Keio University School of Medicine Tokyo, Japan; 2 Department of Endocrinology and Metabolism, Tokyo Metropolitan Children's Medical Center, Tokyo, Japan; 3 Department of Endocrinology and Metabolism, Kanagawa Children's Medical Center, Kanagawa, Japan; Clermont Université, France

## Abstract

Mutations in transcription factors genes, which are well regulated spatially and temporally in the pituitary gland, result in congenital hypopituitarism (CH) in humans. The prevalence of CH attributable to transcription factor mutations appears to be rare and varies among populations.

This study aimed to define the prevalence of CH in terms of nine CH-associated genes among Japanese patients. We enrolled 91 Japanese CH patients for DNA sequencing of *POU1F1, PROP1, HESX1, LHX3, LHX4, SOX2, SOX3, OTX2*, and *GLI2*. Additionally, gene copy numbers for *POU1F1, PROP1, HESX1, LHX3*, and *LHX4* were examined by multiplex ligation-dependent probe amplification. The gene regulatory properties of mutant LHX4 proteins were characterized *in vitro*. We identified two novel heterozygous *LHX4* mutations, namely c.249-1G>A, p.V75I, and one common *POU1F1* mutation, p.R271W. The patient harboring the c.249-1G>A mutation exhibited isolated growth hormone deficiency at diagnosis and a gradual loss of ACTH, whereas the patient with the p.V75I mutation exhibited multiple pituitary hormone deficiency. *In vitro* experiments showed that both *LHX4* mutations were associated with an impairment of the transactivation capacities of *POU1F1* and*αGSU*, without any dominant-negative effects. The total mutation prevalence in Japanese CH patients was 3.3%. This study is the first to describe, a gradual loss of ACTH in a patient carrying an *LHX4* mutation. Careful monitoring of hypothalamic–pituitary -adrenal function is recommended for CH patients with *LHX4* mutations.

## Introduction

The proliferation and terminal differentiation of the anterior pituitary gland is strongly influenced by the precise spatial and temporal expression of transcription factors [1]–[3]. Mutations in these transcription factors often result in various types of congenital hypopituitarism (CH) [1]–[3]. Although previous studies have shown that these transcriptional factor mutations are rare among CH patients and that the mutation prevalence varies among populations, only a few genetic screening studies have been conducted. Graaff *et al*. identified a single patient with a *POU1F1* mutation from a study population of 79 multiple pituitary hormone deficiency (MPHD) patients (1.2%) in The Netherlands [4], and Dateki *et al*. reported one patient harboring an *LHX4* gross deletion from a cohort of 71 MPHD patients (1.4%) in Japan [5]. On the other hand, Reynaud *et al*. reported a mutation prevalence of 13.3% in a study population of 165 MPHD patients from the international GENHYPOPIT network [6]. Approximately 90% of the mutations identified in this report were *PROP1* common mutations (149delGA and 296delGA). Although the 296delGA mutation represents a mutational hot spot within the *PROP1* gene rather than a common founder mutation [7], studies from other ethnic groups often report a low prevalence of *PROP1* mutations [8], [9].

This study aimed to determine the prevalence of transcription factor mutations in Japanese CH patients with PCR-based sequencing of nine CH-associated genes, namely *POU1F1, PROP1, HESX1, LHX3, LHX4, SOX2, SOX3, OTX2*, and *GLI2*. Additionally, we examined the gene copy numbers of *POU1F1, PROP1, HESX1, LHX3*, and *LHX4* by multiplex ligation-dependent probe amplification (MLPA).

## Materials and Methods

### Subjects

This study population consisted of 91 patients with GH-treated CH. The inclusion criteria were as follows: 1) short statue with severe GH deficiency (GH peak < 3 ng/mL) confirmed by hypoglycemic provocation test, and 2) anterior pituitary hypoplasia as detected by brain magnetic resonance imaging (MRI). We excluded any CH patients of known cause, such as a brain tumor or brain surgery from this study. Patients or parents of patients under 18 years of age gave their written informed consent to participate in this study, which was approved by the Institutional Review Board of Keio University School of Medicine and the Institutional Review Board of Kanagawa Children's Medical Center.

### Endocrinological investigations

Hormonal assays were performed using several commercial RIA kits, and normal values for each center were taken into account. The results of biochemical investigations at diagnosis were recorded including basal free thyroxine (fT4), TSH, cortisol and ACTH levels, their peaks in response to pituitary stimulation tests. The patients were evaluated for serum GH level after two consecutive classical provocative tests (with arginine or insulin). GH peaks <6 ng/mL after stimuli support a diagnosis of GHD. GH peak < 3 ng/mL by hypoglycemic provocation test define severe GHD. A diagnosis of TSH deficiency was made if serum fT4 concentration was under the normal level (fT4 < 1.0 ng/dL) with inadequate low serum TSH concentration. Cortisol peaks <17 µg/dL by hypoglycemic provocation tests define ACTH deficiency. FSH–LH deficiency was diagnosed on the basis of delayed or absent pubertal development and inadequate increase in serum FSH and LH in response to LHRH.

### Imaging investigations

MRI included T1 and T2 weighted high-resolution pituitary imaging through the hypothalamo-pituitary axis (T1 sagittal 3-mm slices, T1 and T2 coronal 3-mm slices). Details noted included the size of the anterior pituitary, position of the posterior pituitary signal, presence and morphology of the optic nerves, optic chiasm, pituitary stalk, septum pellucidum, and corpus callosum.

### Mutation screening

For all patients, regardless the phenotype/pituitary MRI findings, we analyzed all coding exons and flanking introns of *POU1F1, PROP1, HESX1*, *LHX3, LHX4, OTX2, SOX2, SOX3*, and *GLI2* by PCR-based sequencing. We screened for deletion/duplication involving *POU1F1, PROP1, HESX1*, *LHX3*, and *LHX4* by MLPA analyses (SALSA MLPA KIT P216; MRC-Holland, Amsterdam, The Netherlands). We tested any detected sequence variations against 150 Japanese control subjects.

### RT-PCR

For mRNA analysis of the *LHX4* c.249-1G>A mutation, total RNA was extracted from Epstein-Barr virus-transformed lymphocytes derived from the propositus of pedigree 1. The cDNA produced from reverse transcription of RNA was subjected to PCR amplification using primers encompassing exons 2 to 4, and were subsequently processed for direct sequencing.

### Functional studies

We performed functional studies on the two novel *LHX4* mutations (p.R84X and p.V75I). To generate LHX4 expression vectors, LHX4 cDNA was cloned into pCMV-myc and pEGFP-N1 (Clontech, Palo Alto, CA). We introduced the two mutations by site-directed mutagenesis, using the PrimeSTAR Mutagenesis Basal Kit (TaKaRa, Otsu, Japan). The luciferase reporter vectors were constructed by inserting the promoter sequences of *POU1F1* (*PIT1*),*αGSU* into a pGL3 basic vector (Promega, Madison, WI). A transactivation assay was performed using dual-luciferase reporter assay system (Promega) on COS7 and GH3 cells. For western blot analyses, we harvested COS7 cells transfected with the myc-tagged LHX4. Western blotting was performed with a mouse anti-myc monoclonal antibody (Invitrogen, Carlsbad, CA). For subcellular localization analyses, we visualized and photographed COS7 cells transfected with GFP-tagged LHX4 using a Leica TCS-SP5 laser scanning confocal microscope (Leica, Exton, PA). The sequences of the biotin-labeled doublestranded oligonucleotide used as probe in the EMSA experiment was 5′-GTATGAATCATTAATTGACAACATATTTTC-3′, as described previously [10]. The probes were detected with the Lightshift chemiluminescent EMSA kit (Pierce) according to the manufacturer's instruction.

## Results

### Patient details

Of the 91 patients, on the basis of hormonal deficiencies, 14 were determined to have isolated GH deficiency (IGHD), whereas 77 were MPHD. Detailed endocrine phenotype was available in all of the 91patients (Table 1). Results of the MRI scans were available in all patients with IGHD and MPHD. Details regarding the structural abnormalities of the hypothalamo-pituitary axis on neuroimaging in the probands are shown in Table 2. Among 77 MPHD patients, 12 were diagnosed as Septo-optic dysplasia.

**Table 1:** Endocrine phenotype of 91 probands screened for 9 genes.

	No. (%) with deficiencies of			
	GH	TSH	ACTH	LH/FSH
IGHD (n = 14)	14(100)			
MPHD (n = 77)	77(100)	61(79)	34(44)	19(24)

**Table 2:** Results of MR scans of probands screened for 9 genes.

	Morphology of						
	Anterior pituitary	Posterior pituitary			Stalk		
	Hypoplasia	Normal	Ectopic	Absent	Normal	Invisible	Thin
IGHD (n = 14)	14	5	9	0	4	5	5
MPHD (n = 77)	77	24	51	2	23	25	29
Total (n = 91)	91	29	60	2	27	30	34

### Mutation screening

**Figure 1:**
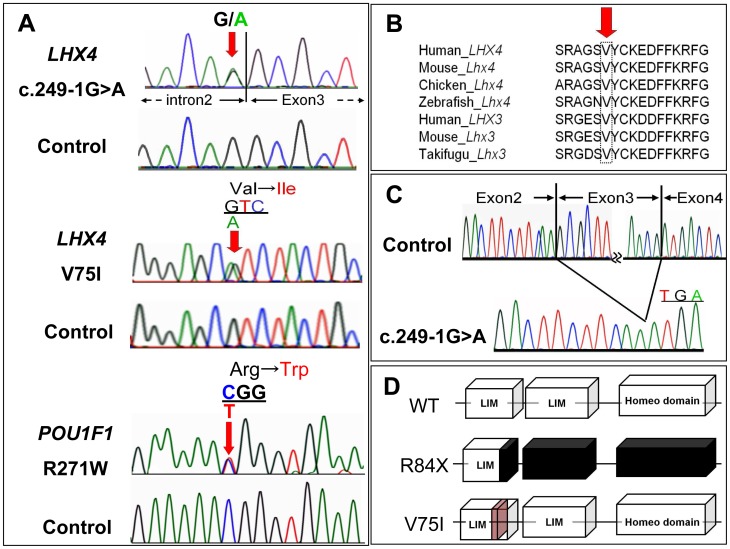
Identification of sequence variations of *LHX4* and *POU1F1*. *A*, Partial sequences of PCR products of the patients are shown. The upper chromatogram represents a heterozygous G to A substitution in the splice acceptor site of exon3. The middle chromatogram represents a heterozygous substitution of isoleucine (ATC) in place of valine (GTC) at codon 75. The arrow indicates the mutated nucleotide. The lower chromatogram represents a heterozygous substitution of tryptophan (TGG) in place of arginine (CGG) at codon 271. The arrow indicates the mutated nucleotide. *B*, Homology study showed valine at codon 75 is highly conserved through species in LHX4 and LHX3. C, Identification of exon3 skipping in the LHX4 cDNA derived from propositus of pedigree 1. LHX4 transcript with a deleted exon 3 creates a premature stop codon at the beginning of the remaining exon 4 (p.R84X). *D*, Schematic diagrams of the LHX4 protein. LHX4 cDNA encodes two LIM domains and one homeodomain. LHX4 with a p.R84X mutation results in the deletion of one of the two LIM domains and the entire homeodomain. Val75 is located within the first LIM domain.

We identified two novel heterozygous *LHX4* mutations, namely c.249-1G>A, expected to cause exon skipping, and c.223G>A (p.V75I), and one common heterozygous *POU1F1* mutation, c.811C>T (p.R271W) [11] (FIG. 1A). The V75 in LHX4 is evolutionarily highly conserved (FIG. 1B), and these two *LHX4* mutations were not detected in any of the 150 healthy Japanese controls. We detected no gross or exon-level deletions/duplications using the MLPA analyses. For 14 IGHD patients, we additionally analyzed all coding exons and flanking introns of *GH1*, and *GHRHR* by PCR-based sequencing and MLPA (SALSA MLPA KIT P216 included all exons of *GH1* and *GHRHR*), failing to detect any sequence variation.

### RT-PCR

The RT-PCR generated a product of smaller size than that obtained from a control sample. Sequencing revealed that it corresponded to a LHX4 transcript skipping exon 3 (FIG. 1C). If translated, this abnormal transcript would generate a protein lacking one of the two LIM domains (LD) and the entire homeodomain (HD), p.R84X (FIG. 1D).

### Clinical phenotypes of the mutation carriers

#### Pedigree 1: *LHX4* c.249-1G>A (FIG. 2A)

The propositus was a 16-year-old Japanese female, who was born at 39 weeks of gestation after an uncomplicated pregnancy and delivery. At birth, her length was 51.0 cm (1.2 SD) and weight 3.3 kg (0.6 SD). She was referred to us at 5 years of age because of short stature. Her height was 92.4 cm (-3.6 SD). Endocrine studies indicated that the patient had IGHD (Table 3). Brain MRI showed anterior pituitary hypoplasia, with a visible but thin stalk, and an ectopic posterior pituitary gland (EPP). No other central nervous system abnormalities were visualized. Recombinant human GH therapy was started at age 6. Her growth was responded well to GH replacement. Although she had no definite episode of adrenal insufficiency, longitudinal data showed that her blood cortisol peak, after stimulation by hypoglycemia with insulin tolerance tests, decreased gradually with age (20.5, 17.5, 16.4, and 10.0 µg/dL, at ages of 5, 13, 14, and 15 years, respectively, Ref. >17 µg/dL [12]), indicating of a gradual loss of ACTH. Follow-up MRI showed no changes as compared with the initial finding.

**Figure 2:**
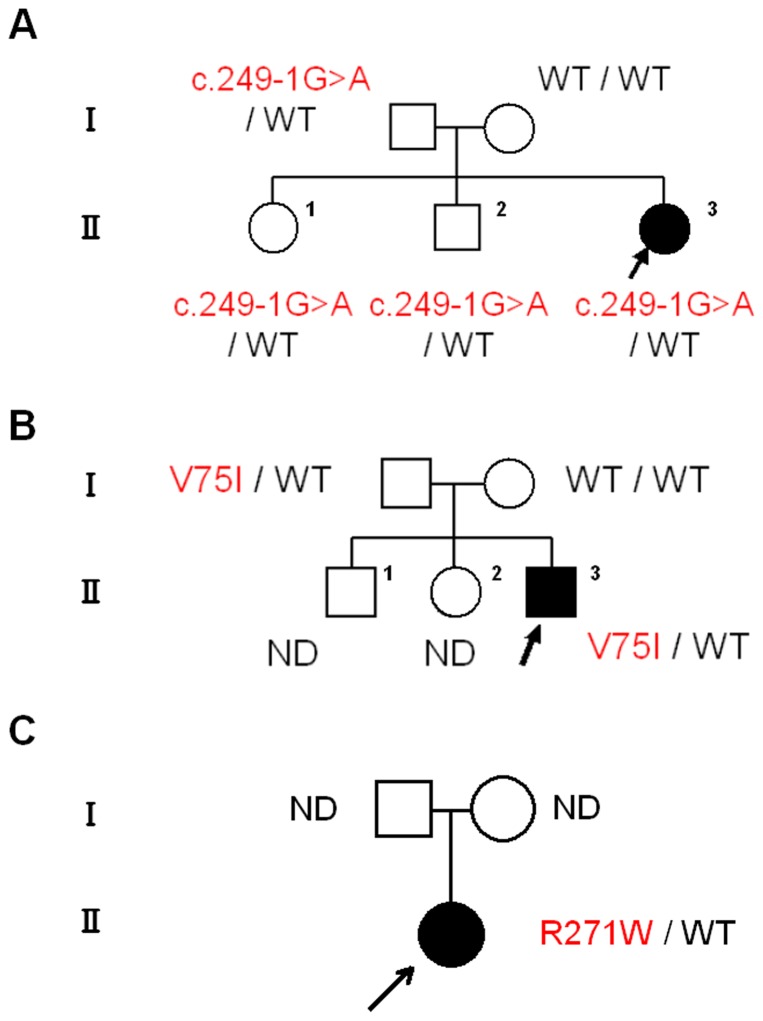
The pedigrees of the affected families. *A–C*, Pedigrees of families 1–3. Arrow indicates the propositus. ND: not determined.

**Table 3:** Endocrinological findings in Propositus of pedigree 1.

		5yr			15yr			Reference	
	Stimulus	Basal		Peak	Basal		Peak	Basal	Peak
GH (ng/ml)	Insulin	2.7	→	2.9	1.8	→	2.6		>6
TSH (mIU/ml)	TRH	2.88	→	10.01	0.78	→	7.42		10–35
LH (mIU/ml)	LHRH	<0.2	→	2.8	6.7	→	21.2	<0.1^a^	1.93–4.73 a
								<0.10–2.65^b^	6.69–22.51 b
FSH (mIU/ml)	LHRH	0.5	→	15.5	7.0	→	9.6	0.64–3.03^a^	13.15–46.95^a^
								1.81–7.31^b^	8.58–17.62^b^
PRL (ng/ml)	TRH	10.4	→	19.7	5.7	→	28.1	1.7–15.4	increase 2 times
ACTH (pg/ml)	Insulin	44	→	46	7.3	→	14.9	9.8–27.3	28–130.5
Cortisol (µg/dl)	Insulin	19.1	→	20.5	7.5	→	10.0		>19.8^c^
									>17.0^d^
IGF-1 (ng/ml)		70.1			241			74–230^e^	
								262–510^f^	
Free T4 (ng/dl)		1.1			1.0			1.0–1.95	
Free T3 (pg/ml)		4.2			2.1			2.23–5.30	
Estradiol (pg/ml)					28			12.3–170^g^	

The conversion factors to the SI unit are as follows: GH 1.0 (µg/liter), LH 1.0 (IU/liter), FSH 1.0 (IU/liter), TSH 1.0 (mIU/liter), prolactin 1.0 (µg/liter), ACTH 0.22 (pmol/liter), cortisol 27.59 (nmol/liter), IGF-I 0.131 (nmol/liter), free T4 12.87 (pmol/liter), free T3, 1.54 (pmol/liter), and estradiol 3.671 (pmol/liter).

a Reference data of pre-pubertal Japanese girls [22]

b Reference data of pubertal (Tanner 2–3) Japanese girls [22]

c Reference data of UK children (younger than 10 years) [23]

d Reference data of UK children (older than 10 years) [23]

e Reference data of Japanese girls (5–7 years old) [24]

f Reference data of Japanese girls (15–17 years old) [24]

g Reference data of Japanese girls (15 years old) [25]

The father of the patient was 153.0 cm (-2.9 SD) tall, and the mother was 160.8 cm (0.5SD) tall. The elder brother and sister of the patient, both reached normal adult heights of 171.7 cm (0.2 SD) and 152.1 cm (-1.3 SD), respectively. Genetic analyses showed that the propositus, siblings and father carried the heterozygous *LHX4* c.249-1G>A mutation. No family members had any baseline hormonal abnormalities (Table 4).

**Table 4:** Endocrinological findings (baseline) in Family members of pedigree 1.

	Father	Mother	Brother	Sister	Reference (Adult)
GH (ng/ml)	0.7	3.2	0.5	0.4	0–23
IGF-1 (ng/ml)	110.0	156.0	357.0	276.0	Male: 41–369
					Female: 73–542
TSH (µU/ml)	0.77	1.60	0.50	0.94	0.3–3.50
Free T4 (ng/dl)	1.1	1.1	1.4	1.3	1.09–2.55
Free T3 (pg/ml)	2.5	2.6	3.1	3.1	3.23–5.11
LH (mIU/ml)	4.8	7.4	2.1	6.9	Male: 2.2–8.4
					Female: 1.4–15^a^
FSH (mIU/ml)	2.9	4.3	2.3	7.9	Male: 1.8–12
					Female: 3–10^a^
PRL (ng/ml)	11.2	11.2	7.8	5.5	Male: 1.5–9.7
					Female: 1.4–14.6
ACTH (pg/ml)	14	12	15	20	7.2–63.3
Cortisol (µg/dl)	8.2	6.3	10.3	10.3	7.6–21.4
Estradiol (pg/ml)		397		23	Female: 11–230^a^
Testosterone (ng/ml)	5.19		5.56		Male: 2.01–7.50

a Follicular phase

#### Pedigree 2: *LHX4* p.V75I (FIG. 2B)

The propositus was a 13-year-old Japanese male born at 41 weeks of gestation after an uncomplicated pregnancy and delivery. At birth, his length was 51.0 cm (1.0 SD) and weight 3.3 kg (0.7 SD). He was referred to us at 3 months of age because of a micropenis and bilateral cryptorchidism. He had undetectable plasma testosterone and LH levels, indicating hypogonadotropic hypogonadism. Severe growth failure was observed at the age of 11 months. Hormonal data revealed GH and TSH deficiencies in addition to tentative gonadotropin deficiency (Table 5). Brain MRI exhibited anterior pituitary hypoplasia, poorly developed sella turcica, visible but thin stalk, and EPP. No other central nervous system abnormalities were visualized. Replacement therapy with thyroxine and recombinant human GH was started at the age of 1 year. The patient responded well to GH replacement. At the age of 13 years, he showed small intrascrotal testes (1 ml), no pubic hair (P1), and a microphallus with low concentration of basal testosterone (0.05 ng/mL Ref: 2.0–7.5).

**Table 5:** Endocrinological findings in Propositus of pedigree 2.

		11month			8yr			Reference	
	Stimulus	Basal		Peak	Basal		Peak	Basal	Peak
GH (ng/ml)	Insulin	1.1	→	0.9	0.6	→	0.6		>6
TSH (mIU/ml)	TRH	0.56	→	6.81	2.00	→	10.81		10-35
LH (mIU/ml)	LHRH	0.3	→	0.8	0.2	→	2.3	<0.1^a^	<0.10-4.29 a
FSH (mIU/ml)	LHRH	2.1	→	2.6	1.5	→	7.4	0.46-1.43^a^	5.38-11.67^a^
Testosterone (ng/ml)	HCG				<0.05		0.17		>1.2^a^
PRL (ng/ml)	TRH	5.6	→	10.1	7.7	→	13.0	1.7-15.4	increase 2 times
ACTH (pg/ml)	Insulin	44	→	170	44	→	50	9.8-27.3	28-130.5
Cortisol (μg/dl)	Insulin	31.0	→	38.4	13.4	→	17.2	5-20	>19.8^b^
IGF-1 (ng/ml)		6.9			157			18-150 c	
								50-356^d^	
Free T4 (ng/dl)		1.1			1.1			1.01-1.95	
Free T3 (pg/ml)		4.4			3.9			2.23-5.30	

The conversion factors to the SI unit are as follows: GH 1.0 (μg/liter), TSH 1.0 (mIU/liter), LH 1.0 (IU/liter), FSH 1.0 (IU/liter), testosterone, 0.035 (nmol/liter), prolactin 1.0 (μg/liter), ACTH 0.22 (pmol/liter), cortisol 27.59 (nmol/liter), IGF-I 0.131 (nmol/liter), free T4 12.87 (pmol/liter), and free T3, 1.54 (pmol/liter).

a Reference data of pre-pubertal Japanese boys (younger than 10 years) [22]

b Reference data of UK children (younger than 10 years) [23]

c Reference data of Japanese boys (younger than 1 years old) [24]

d Reference data of Japanese boys (7-9 years old) [24]

The patient's father was 160.5 cm (-1.8 SD) tall. Genetic analyses showed that the propositus and father carried the same heterozygous *LHX4* p.V75I mutation. No other family member was available for genetic studies. Evaluation of the hormonal data for the father was refused.

#### Pedigree 3: *POU1F1* p.R271W (FIG. 2C)

The propositus was a 28-year-old Japanese female, who was born at 37 weeks of gestation after an uncomplicated pregnancy and delivery. At birth, her length was 48.0 cm (-0.2 SD) and weight 2.6 kg (-1.0 SD). She was referred to us at 2 years of age because of severe short stature (-4.5 SD). Endocrine studies indicated that the patient had complete GH and PRL deficiencies and partial TSH deficiency (free T4 0.8 ng/dl, Ref. >1.0, with inadequate low TSH). Brain MRI at the age of 7 years exhibited anterior pituitary hypoplasia, normal stalk, and normal posterior pituitary gland. No other central nervous system abnormalities were visualized. The patient responded well to GH replacement.

### Functional studies

**Figure 3:**
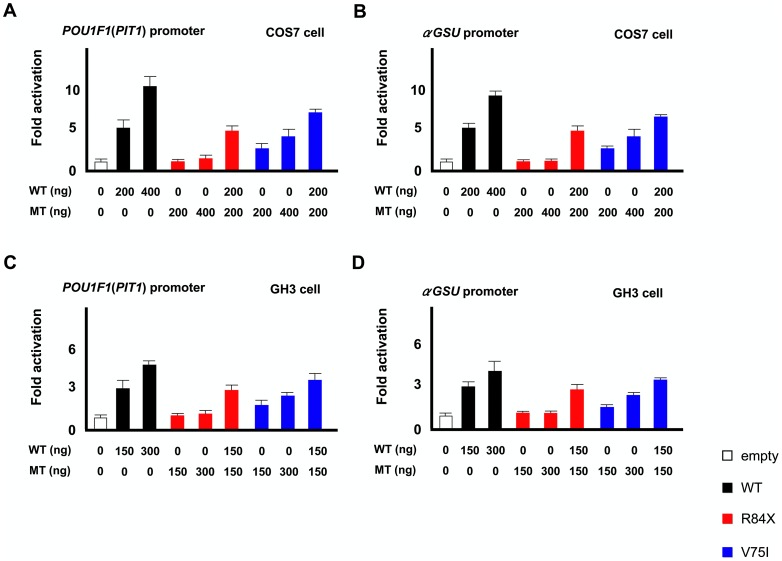
Transactivation assays of R84X and V75I LHX4 using *POU1F1*(*PIT1*) and*αGSU* reporter. *A and B*: COS7 cells were cotransfected with the pRL-CMV internal control vector, indicated amount (nanograms) of the effector plasmids, and the *POU1F1*(A) or*αGSU* (B) reporter. The data are the mean ± s.e.m. of at least three independent experiments performed in triplicate transfections. The white, black, red, and blue bars indicate the data of the empty expression vectors, expression vectors with wild type (WT) LHX4, expression vectors with R84X LHX4, and V75I LHX4, respectively. R84X LHX4 exhibited markedly reduced transactivation, whereas V75I LHX4 retained partial activity. The two mutants did not exhibit any dominant negative effect. The data are mean ± SEM of at least three independent experiments performed in triplicate transfections. *C and D*: GH3 cells were cotransfected with the pRL-CMV internal control vector, indicated amount (nanograms) of the effector plasmids, and the *POU1F1*(C) or*αGSU* (D) reporter.

**Figure 4:**
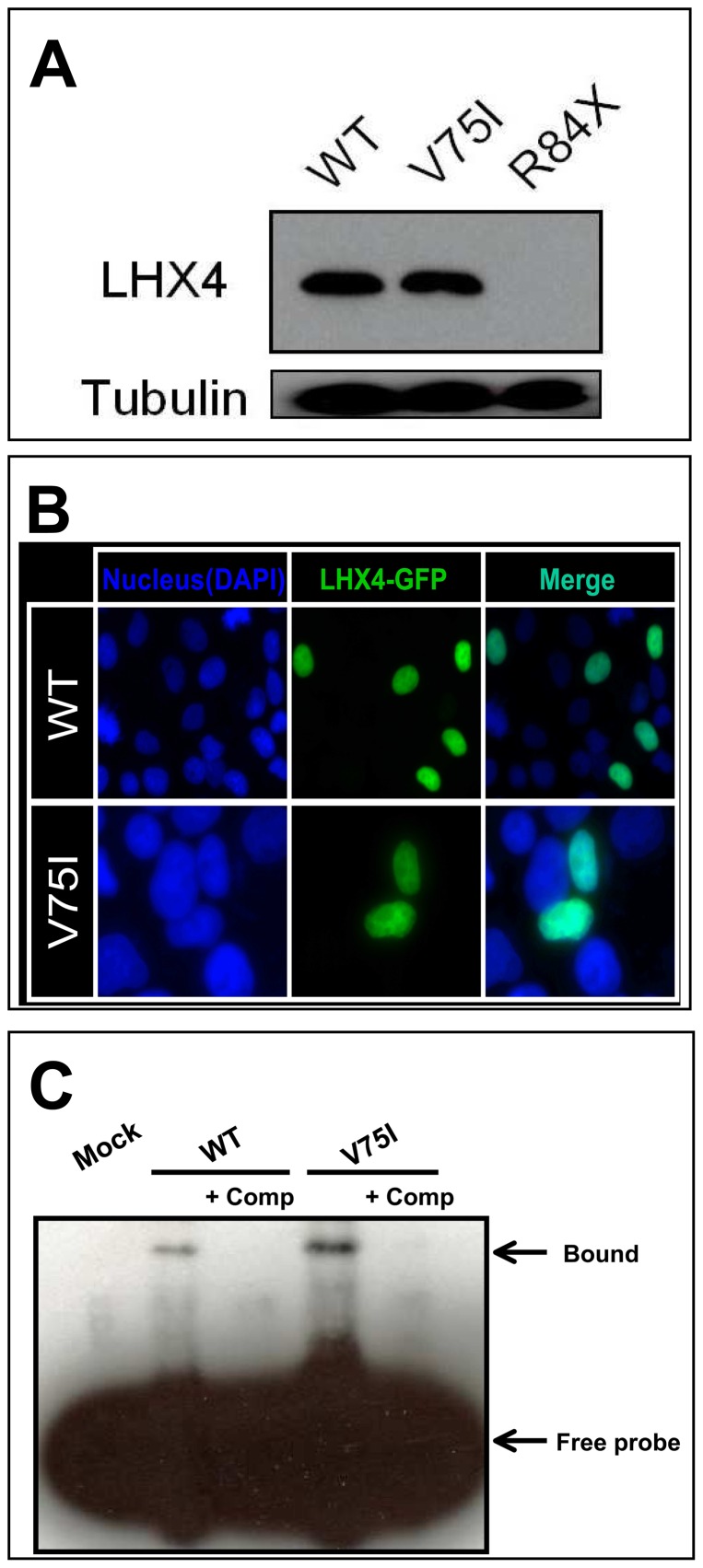
Functional characterization of two mutant LHX4. *A*, Protein expression level of myc-tagged WT and two LHX4 mutants was assessed by western blot using a monoclonal anti-myc antibody. The expression of V75I LHX4 was comparable to that of WT, whereas R84X LHX4 was not detected. Tubulin was used as a control. *B*, Subcellular localization analysis. For subcellular localization analyses, we visualized and photographed COS7 cells transfected with GFP-tagged LHX4 using a Leica TCS-SP5 laser scanning confocal microscope, after mounting the cells in Vectashield-DAPI solution. The WT and V75I LHX4 are localized to the nucleus. *C*, EMSA experiments. WT LHX4 showed specific binding to the elements, which was competed by excess amount of (200 times) cold competitors. The V75I LHX4, which has an intact HD, bound with similar or slightly high efficiency to the WT LHX4.

Both in COS7 and GH3 cells, wild type LHX4 stimulated transcription of the *POU1F1* and*αGSU* reporters in a dose-dependent manner. R84X LHX4 had markedly reduced transactivation, whereas V75I LHX4 retained partial activity (FIG. 3A-D). The two mutants had no dominant negative effect. Western blot analysis showed that the expression of V75I LHX4 was comparable to that of the wild type, whereas R84X LHX4 was not detected (FIG. 4A). The V75I LHX4 mutant localized to the nucleus (FIG. 4B). WT LHX4 showed specific binding to the elements, which were competed by excess amount of (200 times) cold competitors. The V75I LHX4, which has an intact HD, bound with similar or slightly high efficiency to the WT LHX4 (FIG. 4C).

## Discussion

In the present study, our mutation prevalence data (three mutation carriers in a total of 91 CH patients: 3.3%) is comparable with earlier report of Graaff *et al*. (1.2%) [4] or Dateki *et al* (1.4%) [5]. This study enrolled CH patients that fulfilled two definite inclusion criteria: 1) severe GH deficiency (GH peak < 3 ng/mL) confirmed by hypoglycemic provocation tests, which included IGHD and MPHD, and 2) anterior pituitary hypoplasia based on brain MRI. The subjects included in the two previous reports were diagnosed with MPHD and the reports of Dateki *et al*. did not describe any specific inclusion criteria. As *PROP1* common mutations (149delGA and 296delGA) are rare in Japan, our prevalence data were lower than that of Reynaud *et al*. [6]. These previous studies did not include screening for *SOX2*, *SOX3*, *OTX2* and *GLI2* (although the study by Dateki *et al*. included *SOX3* and *OTX2*), thus this study serves as the first report to include these genes. Despite extending the range of our genetic screening, our results imply the rarity of pathological abnormalities in the currently known genes responsible for CH. Further studies are required to understand the pathogenesis of CH.

To date, eight families carrying a *LHX4* mutation have been reported [5], [13]–[17]. We identified two novel mutations in *LHX4* (c.249-1G>A, p.V75I). Although both mutations were associated with impaired transactivation of *POU1F1* and*αGSU* without dominant-negative effects, indicating haploinsufficiency, the mechanism behind the loss of function resulting from these two mutations seems to be different. We did not detect R84X LHX4 on western blotting, indicating that the protein expression is markedly reduced due to the protein's instability. On the other hand, western blotting, visualization of subcellular localization, and DNA binding test revealed no significant difference between the wild type and V75I LHX4 variant. Val75 is a highly conserved amino acid located in the LD (FIG. 1C), which is important for protein-protein interaction, suggesting that substitution of Val75 to Ile results in defective interactions with transcriptional cofactors.

A striking finding of our report is that the propositus, who carried the c.249-1G>A *LHX4* mutation, exhibited a gradual loss of ACTH. Although late onset ACTH deficiency is well known in CH patients with *PROP1* mutations [18]–[20] and LHX3 [21], our study showed, for the first time, that a gradual loss of ACTH should be a point of concern among CH patients with *LHX4* mutations. Thus, this study suggests careful follow-up monitoring of the hypothalamic-pituitary-adrenal function in CH patients with *LHX4* mutations even if ACTH deficiency is not apparent at first evaluation. The patient's elder brother and sister were of normal adult height and had normal baseline hormonal levels. Even though this report is not the first description of the wide phenotypic spectrum in *LHX4* mutation carriers [13]–[17], it is noteworthy that *LHX4* mutation carriers can clinically and endocrinologically present as normal, even though the mutation is nonfunctional. The phenotypical variation documented in this study for patients with MPHD with mutations in *LHX4*, including dissimilarity within probands from the same pedigree, is likely partly due to the impact of other genes that are important but have not been recognized in pituitary development.

In summary, we found that only 3.3% of Japanese patients had mutation. *LHX4* mutation carriers exhibit wide phenotypic variability and can present as normal clinically and endocrinologically, even though they had a nonfunctional mutation. Gradual loss of ACTH should be monitored in CH patients with *LHX4* mutations.
